# NQO1 overexpression is associated with poor prognosis in squamous cell carcinoma of the uterine cervix

**DOI:** 10.1186/1471-2407-14-414

**Published:** 2014-06-09

**Authors:** Yue Ma, Jienan Kong, Guanghai Yan, Xiangshan Ren, Dan Jin, Tiefeng Jin, Lijuan Lin, Zhenhua Lin

**Affiliations:** 1 Department of Pathology & Cancer Research Center, Yanbian University Medical College, Yanji, China; 2 Department of Anatomy and Histology and Embryology, Yanbian University Medical College, Yanji, China; 3 Department of Internal Medicine, Yanji City Hospital, Yanji, China; 4 Department of Medical Imaging, College of Medicine, Eastern Liaoning University, Dandong, China

**Keywords:** Squamous cell carcinoma, Cervix uteri, NQO1, Human papillomavirus, Prognosis, Survival analysis

## Abstract

**Background:**

NQO1 (NAD(P)H: quinone oxidoreductase-1), located on chromosome 16q22, functions primarily to protect normal cells from oxidant stress and electrophilic attack. Recent studies have revealed that NQO1 is expressed at a high level in most human solid tumors including those of the colon, breast, pancreas, ovaries and thyroid, and it has also been detected following the induction of cell cycle progression and proliferation of melanoma cells. In this study, we aimed to investigate the clinicopathological significance of upregulated NQO1 protein expression in squamous cell carcinomas (SCCs) of the uterine cervix.

**Methods:**

The localization of the NQO1 protein was determined in the SiHa cervical squamous cancer cell line using immunofluorescence (IF) staining, and immunohistochemical (IHC) staining performed on paraffin-embedded cervical SCC specimens from 177 patients. For comparison, 94 cervical intraepithelial neoplasia (CIN) and 25 normal cervical epithelia samples were also included. QRT-PCR was performed on RNA from fresh tissues to detect NQO1 mRNA expression levels, and HPV infection status was genotyped using oligonucleotide microarray. Disease-free survival (DFS) and 5-year overall survival (OS) rates for all cervical SCC patients were calculated using the Kaplan–Meier method, and univariate and multivariate analysis was performed using the Cox proportional hazards regression model.

**Results:**

The NQO1 protein showed a mainly cytoplasmic staining pattern in cervical cancer cells, and only three cases of cervical SCC showed a nuclear staining pattern. The strongly positive rate of NQO1 protein expression was significantly higher in cervical SCCs and CINs than in normal cervical epithelia. High-level NQO1 expression was closely associated with poor differentiation, late-stage, lymph node metastasis and high-risk for HPV infection. Additionally, high-level NQO1 expression was associated with lower DFS and 5-year OS rates, particularly for patients with early-stage cervical SCCs. Furthermore, Cox analysis revealed that NQO1 expression emerged as a significant independent hazard factor for DFS rate in patients with cervical SCC.

**Conclusions:**

NQO1 overexpression might be an independent biomarker for prognostic evaluation of cervical SCCs.

## Background

Uterine cervical cancer has been estimated to affect 500,000 women annually and cause 270,000 deaths worldwide [1]. It is characterized by a range of minor to severe neoplastic changes in the epithelium, which typically advances locally and via the lymphatic route, sometimes recurrently [2]. Advances in therapeutic methods and diagnostic tools have not improved the overall prognosis of patients with recurrent cervical cancer, and optimal treatment for recurrent disease is still open to debate [3]. Therefore, understanding the molecular events and mechanisms underlying tumor initiation and progression, which could contribute to early detection, will be helpful in the prevention and treatment of cervical cancer.

The gene for NAD(P)H: quinone oxidoreductase-1 (NQO1), also known as DT-diaphorase, is located on chromosome 16q22 and consists of six exons and five introns [4]. NQO1 is mainly a cytosolic enzyme that uses NADH or NADPH as substrates to catalyze the two-electron reduction of quinines to their hydroquinone forms [5], thus bypassing toxic semiquinone intermediates, and these resultant hydroquinones are thus ready for further conjugation and excretion [6]. However, conclusions on the biological functions of NQO1 in cancer have been contradictory. On the one hand, NQO1 is induced along with a battery of defensive genes that provide protection against different stresses to prevent organs from undergoing carcinogen-induced tumorigenesis. On the other hand, reductive activation of environmental carcinogens including dinitropyrenes and heterocyclic amines by NQO1 could contribute to carcinogenesis [7]. Interestingly, NQO1 was found to be expressed at high levels in many solid tumors, including cholangiocarcinoma [8], lung [9] and pancreas [10], and has also been detected following the induction of cell cycle progression and proliferation of melanoma cells [11]. To date, the correlation between NQO1 expression and cervical cancer has not been adequately studied.

In this study, we aimed to analyze the expression status of NQO1 in squamous cell carcinomas (SCCs) of the uterine cervix, normal cervical epithelia and precancerous disease, investigate the relationship between it and clinicopathological parameters and discover its prognostic value in cervical SCC patients based on survival data.

## Methods

### Ethics statement

This study complied with the Helsinki Declaration and was approved by the Human Ethics Committee and the Research Ethics Committee of Yanbian University Medical College of China. Through the surgery consent form, patients were informed that the resected specimens were kept by our hospital and might be used for scientific research, and that their privacy would be maintained. Follow-up survival data were collected retrospectively through medical-record analyses.

### Tissue specimens

The routinely processed and diagnosed uterine cervical lesions, including 25 non-neoplastic cervical tissues, 94 cervical intraepithelial neoplasias (CIN; CIN-1, n = 29; CIN-2, n = 38; CIN-3, n = 27) and 177 SCCs were selected randomly from patients undergoing surgery between January 2004 and November 2008 and stored in the Tumor Tissue Bank of Yanbian University Medical College. All cervical tissue specimens were selected from punch biopsies, loop electrosurgical excisions, cone biopsies and hysterectomies, and all 25 non-neoplastic cervical tissues were obtained from leiomyoma patients with hysterectomies. The cancer patients were aged 22–76 years. All SCC tumor specimens were obtained from pretreatment surgical resections, and the data were retrieved from patients’ operative and pathological reports, while follow-up data were obtained by phone and from an outpatient clinical database. The SCC patients with lymph node metastasis were defined by the clinical and/or radiological investigation of significantly enlarged lymph node, or lymph node biopsy. Staging was performed according to the TNM and FIGO classification of carcinoma of the uterine cervix, and 101 tumors were FIGO stage I-IIA (early stage) and 76 were stage IIB-IV (advanced stage), according to the Union for International Cancer Control (UICC) criteria 7th Edition and World Health Organization classification [12]. All cervical cancer patients had follow-up records for more than 5 years, and the follow-up deadline was January 2013. Survival time was counted from the date of surgery to the follow-up deadline, or date of death (usually the result of cancer recurrence or metastasis). Two experienced pathologists (Lin Z, Ren X) reviewed the H&E stained slides, after which one appropriate paraffin block for each sample was selected for this study. The Human Ethics Committee and the Research Ethics Committee of Yanbian University approved the study protocol.

### IHC analysis

Tissue array for partial cases were produced by Shanghai Outdo Biotech Co.,Itd, China. IHC analysis was performed using the DAKO LSAB kit (DAKO A/S, Glostrup, Denmark). Briefly, to eliminate endogenous peroxidase activity, tissue sections were deparaffinized, rehydrated and incubated with 3% H_2_O_2_ in methanol for 15 min at room temperature (RT). Antigen retrieval was performed at 95°C for 20 min by placing the slides in 0.01 M sodium citrate buffer (pH 6.0). The slides were then incubated with the NQO1 antibody (1:200, Santa Cruz Biotechnology, Dallas, TX, USA) at 4°C overnight. After incubation with the biotinylated secondary antibody at RT for 30 min, the slides were incubated with streptavidin-peroxidase complex at RT for 30 min. Immunostaining was developed using 3,3′-diaminobenzidine, and Mayer’s hematoxylin was used for counterstaining [13]. Mouse IgG was used as an isotope control. In addition, positive tissue sections were processed while omitting the primary antibody (mouse anti-NQO1) as negative controls.

All specimens were blind examined by two pathologists (Lin Z and Jin T). In case of discrepancies, a final score was established by reassessment on a double-headed microscope. Interpretation criteria were as previously described [14]. Briefly, The immunostaining for NQO1 was semi-quantitatively scored as ‘-’ (negative) no or less than 5% positive cells; ‘+’ 5–25% positive cells; ‘++’ 26–50% positive cells; and ‘+++’ more than 50% positive cells. Either cytoplasmic or nuclear NQO1 expression pattern was considered as positive staining, and ++ or +++ scored samples were considered as strongly positive. For survival data analysis, ++ or +++ scored samples were considered as high-level and – or + scored samples were considered as low-level NQO1 expression.

### HPV genotyping by oligonucleotide microarray (HPV-DNA chip)

DNA was extracted from paraffin-embedded cervical lesion specimens using the High Pure PCR Template Preparation kit (Cat.11796828001, Roche, Penzberg, Germany), and HPV detection and genotyping were performed using a PCR-based HPV-DNA microarray system (Biomedlab, Seoul, South Korea). HPV-DNA chips can detect 22 types of HPV, including 15 high-risk types (HPV16, 18, 31, 33, 35, 39, 45, 51, 52, 56, 58, 59, 66, 68 and 69) and seven low-risk types (6, 11, 34, 40, 42, 43 and 44). Target HPV-DNA was amplified using PCR with the following primers (forward, 5′-TTTKTTACHGTKGTDGATACYAC-3′; reverse, 5′-GAAAHATAAAYTGYAADTCATAYTC-3′; K, G/T; H, T/A/C; D, A/T/G; Y, T/C) and labeled with Cy5-dUTP (NEN, life Science Products, MA, USA). A 110 bp β-globin fragment was amplified as an internal control. The assay was performed according to the manufacturer’s protocol (GSI Lumonics, ScanArray Lite, Ottawa, ON, Canada), as described previously [15].

### IF staining of the NQO1 protein in SiHa SCC cells

The cervical SCC cell line, SiHa, was grown on coverslips to 70% confluence and the cells were then fixed in 4% paraformaldehyde for 10 min and permeabilized with 0.5% TritonX-100 for 10 min after 24 h. Blocking was performed with 3% bovine serum albumin fraction V (A8020, Solarbio, Beijing, China) for 1 h at RT. After washing with phosphate-buffered saline (PBS), cells were incubated with anti-rabbit NQO1 (1:200, Santa Cruz Biotechnology) at 4°C overnight, followed by incubation with Alexa Fluor^®^ 568 goat anti-mouse IgG (H + L) (A11004, 1:1000, Life Technologies, Carlsbad, CA, USA), respectively, for 1 h at RT. After washing with PBS, cells were counterstained with 49-6-diamidino-2-phenylindole (DAPI) (C1006, Beyotime, Shanghai, China) and the coverslips were mounted with Antifade Mounting Medium (P0126, Beyotime, Shanghai, China). Finally, IF signals were visualized and recorded using a Leica SP5II confocal microscope [16].

### RNA extraction and qRT-PCR

Total RNA was extracted from 8 fresh samples of normal cervical epithelia and 12 SCC samples using the TRIzol Reagent (Invitrogen, Carlsbad, CA, USA), as described previously [14]. We used PrimeScript reverse transcriptase (Takara Biotechnology, Dalian, China) and oligo(dT) to synthesize the first-strand cDNA according to the manufacturer’s instructions. QPCR was performed using a double-stranded DNA-specific SYBR Premix Ex Taq™ II kit (Takara Biotechnology) on a Bio-Rad sequence detection system according to the manufacturer’s instructions. Double-stranded DNA-specific expression was quantified using the comparative Ct method (2-ΔΔCt). NQO1 primers used were: 5'-GGCAGAAGAGCACTGATCGTA-3', and 5'-TGATGGGATTGAAGTTCATGGC-3'. Primers for GAPDH, which was used as an internal control, were: 5’-GGTCTCCTCTGACTTCAACA-3’ and 5’-ATACCAGGAAATGAGCTTGA-3’. All assays were performed in triplicate and repeated at least three times.

### Statistical analysis

The chi-square test was used to analyze the univariate associations of clinicopathological features with the expression status of NQO1. Survival curves were calculated using the Kaplan–Meier method, and differences were analyzed using the log-rank test. Multivariate analysis was performed using the Cox proportional hazards regression model on all significant characteristics determined using univariate analysis. P-values of less than 0.05 were considered statistically significant. All analyses were performed using the SPSS 17.0 statistical package (SPSS, Inc., Chicago, IL, USA).

## Results

### High-level expression of NQO1 in cervical cancers

**Figure 1:**
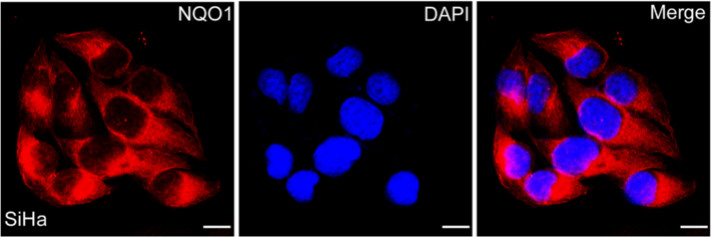
**Immunofluorescence staining of the NQO1 protein in SiHa cervical SCC cells.** The NQO1 protein was located in the cytoplasm of SiHa cervical SCC cells (red indicates NQO1 staining, blue indicates DAPI).

**Figure 2:**
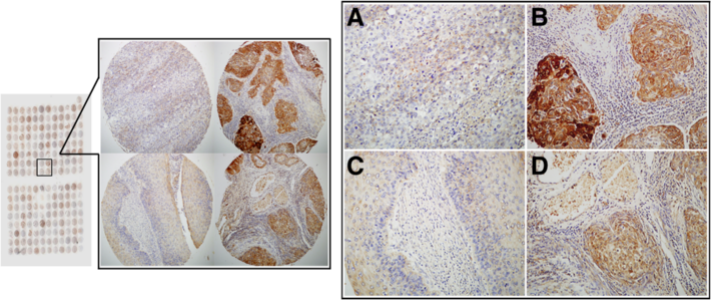
**IHC staining of the NQO1 protein on tissue microarray of cervical lesions. (A)** NQO1 protein was weakly positive in cervical SCC. **(B)** NQO1 protein showed diffuse and strong cytoplasmic-positive staining in cervical SCC. **(C)** NQO1 protein was negative in normal cervical epithelia. **(D)** NQO1 protein was positive in SCC.

**Figure 3:**
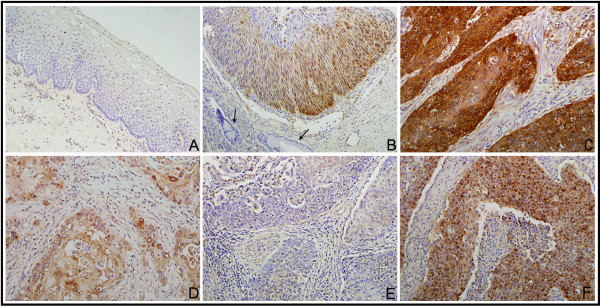
**NQO1 protein expression in cervical lesions using IHC. (A)** NQO1 protein was negative in normal cervical epithelia. **(B)** NQO1 protein staining was positive in dysplastic cells of CIN-3, but negative in adjacent normal cervical glands (*arrows*). **(C)** NQO1 protein showed diffuse and strong cytoplasmic-positive staining in late-stage cervical SCC. **(D)** NQO1 was weakly positive in early-stage cervical SCC. **(E)** NQO1 protein was negative in cervical SCC without metastasis. **(F)** This case of SCC showed a rare nuclear staining pattern.

IF staining revealed that the NQO1 protein was located in the cytoplasm of SiHa SCC cells (Figure 1). IHC staining consistently showed that the NQO1 protein was mainly cytoplasmic (Figure 2), while only three cases showed nuclear staining in SCCs (Figure 3F). The positive rate of NQO1 protein expression was only 12.00% (3/25) in non-neoplastic cervical tissues, but significantly higher in CIN lesions (41.38% in CIN-1, 52.63% in CIN-2 and 55.56% in CIN-3) and SCCs (80.23%, 142/177) of the cervix (*P* < 0.01). Additionally, the strongly positive rates of NQO1 expression were also significantly higher in CINs (27.59% in CIN-1, 34.21% in CIN-2 and 40.74% in CIN-3) and SCCs (54.80%) of the cervix than in the normal cervical epithelia (4%, 1/25) (*P* < 0.01) (Figures 2 and 3, Additional file 1: Table S1).

**Figure 4:**
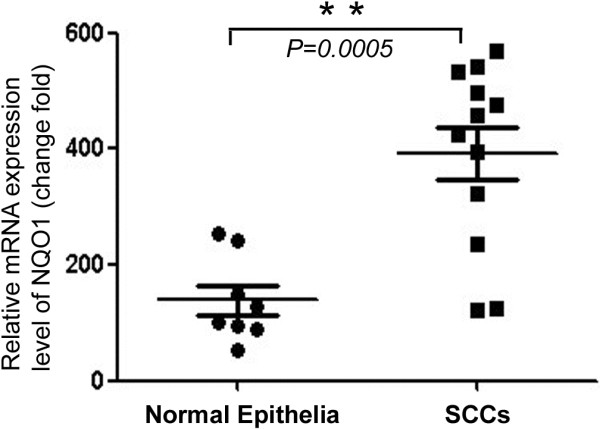
**QRT-PCR analysis of NQO1 mRNA.** Twelve cases of cervical SCC and eight cases of normal fresh cervical tissue were collected and subjected to qRT-PCR analysis of NQO1 mRNA levels. Data represent the mean of individual samples tested in triplicate relative to that of the normal control ± SD (*P* < 0.05).

QRT-PCR data also confirmed increased levels of NQO1 mRNA expression in cervical SCCs compared with fresh samples of normal cervical epithelium (Figure 4).

### Correlation between high-level NQO1 expression and clinicopathological parameters of cervical SCCs

NQO1 protein high-level expression rate was significantly higher in poorly (65.22%, 30/46) and moderately (58.21%, 39/67) differentiated cervical SCCs than in well-differentiated cervical SCCs (43.75%, 28/64) (*P* = 0.022). It was also higher in cervical SCCs with lymph node metastasis (65.38%, 51/78) than in cases without metastasis (46.46%, 46/99) (*P* = 0.012). For the TNM and FIGO clinical stages, the strongly positive rate of NQO1 protein expression was 69.74% (53/76) in advanced (stages IIB–IV) cervical SCCs, but only 43.56% (44/101) in early stage cases (I–IIA) (*P* = 0.001). However, high-level NQO1 expression was not related to age (*P* > 0.05) (Table 1).

**Table 1:** Correlation between NQO1 expression and clinicopathological features in 177 patients with SCC

**Clinical features**	**NQO1 positive cases (%)**		**χ**^**2**^	***P*-value**
	**−/+**	**++/+++**		
**Age**				
≤45	32 (47.76%)	35 (52.24%)	0.286	0.594
>45	48 (43.64%)	62 (56.36%)		
**Stage**				
I-IIa	57 (56.44%)	44 (43.56%)	11.993	0.001**
IIb-IV	23 (30.26%)	53 (69.74%)		
**Differentiation**				
Well	36 (56.25%)	28 (43.75%)		
Moderate	28 (41.79%)	39 (58.21%)	5.485	0.022*
Poorly	16 (34.78%)	30 (65.22%)		
**Lymph node metastasis**				
Negative	53 (53.54%)	46 (46.46%)	6.305	0.012*
Positive	27 (34.62%)	51 (65.38%)		

* *P* < 0.05, ** *P* < 0.01.

### Correlation between high-level NQO1 expression and high-risk HPV infection in cervical SCCs

All 25 cases of normal cervical epithelium were negative for high-risk HPV infection. The positive rate of high-risk HPV infection was 65.52% in CIN-1, 76.32% in CIN-2, 74.07% in CIN-3 and 86.44% in cervical SCCs according to HPV-DNA PCR detection. Of 177 cases of cervical SCC, 153 cases were positive for high-risk HPV infection and 24 cases were negative. Interestingly, the strongly positive rate of NQO1 protein expression was significantly higher in HPV-infected cervical SCCs (58.17%, 89/153) than in HPV-negative cases (33.33%, 8/24) (P = 0.023). This result may indicate a positive correlation between high-risk HPV infection and high-level NQO1 expression in cervical SCCs. However, a statistically significant correlation between high-level NQO1 expression and high-risk HPV infection in precancerous lesions was not found (Additional file 1: Table S2).

### High-level NQO1 expression predicts poor survival rates in patients with cervical SCC using the Kaplan–Meier method

**Figure 5:**
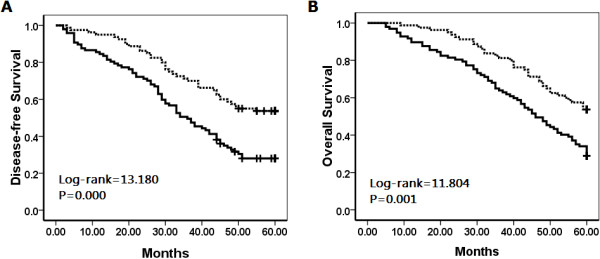
**Kaplan–Meier analysis of cervical SCC patient survival rates in relation to NQO1 protein expression.** Disease-free survival **(A)** and overall survival rates **(B)** of patients with elevated (solid, n = 97) and low (dashed, n = 80) NQO1 expression.

To further confirm the role of NQO1 expression in cervical SCC progression, we analyzed disease-free survival (DFS) and overall survival (OS) rates for 177 cervical SCC cases using the Kaplan–Meier method, and found that patients with high-level NQO1 expression had lower DFS (log-rank = 13.180, *P*<0.001) and 5-year OS rates (log-rank = 11.804, *P* = 0.001) than those with low-level NQO1 expression (Figure 5).

**Figure 6:**
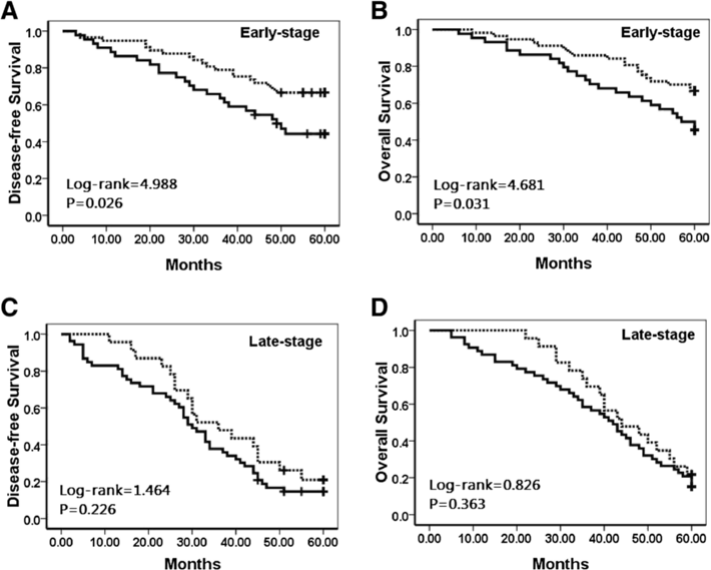
**Kaplan–Meier analysis of survival rates in patients with high- or low-level NQO1 expression and with early- or late-stage cervical SCC.** Disease-free survival **(A)** and overall survival rates **(B)** were assessed in patients with early-stage cervical SCC concomitant with either high- (solid, n = 44) or low-level (dashed, n = 57) NQO1 expression. Disease-free survival **(C)** and overall **(D)** survival rates were also assessed in patients with late-stage cervical SCC concomitant with high- (solid, n = 53) or low-level (dashed, n = 23) NQO1 expression.

To further substantiate the importance of NQO1 expression in cervical SCC progression, we analyzed the correlation between the high-level NQO1 expression rate and clinical stages of cervical SCCs. In early-stage cervical SCCs, patients with high-level NQO1 expression had lower DFS and 5-year OS rates compared with those with low-level NQO1 expression (*P* = 0.026 and *P* = 0.031, respectively) (Figure 6A–B). We also found that the patients with high-level NQO1 expression have lower DFS and 5-year OS rates in either stage-IA1-2 or stage-IB-IIA SCC patients, however, there were no statistical significances (data not shown). Additionally, in patients with late-stage cervical SCC, DFS and 5-year OS rates were not correlated with NQO1 expression status (*P* = 0.226 and *P* = 0.363, respectively) (Figure 6C–D).

### NQO1 is an independent prognostic factor for survival of cervical SCCs using the Cox proportional hazards regression model

On univariate analysis, patients with high-level NQO1 expression cervical SCC tumors had significantly lower DFS and OS rates (*P* = 0.001 and *P* = 0.027, respectively) than those with low-level NQO1 expression tumors. Additionally, clinical stage and lymph node metastasis were also associated with DFS and 5-year OS rates of cervical SCC patients. Therefore, multivariate survival analysis was then performed using the Cox proportional hazards regression model for all significant variables identified in the univariate survival analysis. We found that clinical stage and lymph node metastasis proved to be independent prognostic factors for DFS and 5-year OS rates in cervical SCC patients. Importantly, NQO1 expression also emerged as a significant independent prognostic factor for DFS in patients with cervical SCCs (HR: 1.387, 95% CI: 1.017-1.890, *P* = 0.039). However, it was not a significant independent prognostic factor for 5-year OS (HR: 1.154, 95% CI: 0.845-1.576, *P* = 0.368) (Table 2).

**Table 2:** Univariate and multivariate survival analyses (Cox regression model) of various factors in 177 patients with SCC

**Factors**	**DFS Hazard ratio (95% CI)**	***P*-value**	**OS Hazard ratio (95% CI)**	***P*-value**
**Univariate analyses**				
Age	1.107 (0.752-1.375)	0.912	1.052 (0.779-1.423)	0.740
HPV	1.119 (0.769-1.627)	0.557	1.011 (0.696-1.470)	0.953
Differentiation	1.206 (0.992-1.467)	0.061	1.181 (0.975-1.431)	0.090
Stage	2.110 (1.552-2.870)	0.000**	1.885 (1.391-2.553)	0.000**
Lymph node	1.706 (1.260-2.310)	0.001**	1.597 (1.182-2.157)	0.002**
NQO1	1.649 (1.222-2.224)	0.001*	1.397 (1.038-1.880)	0.027*
**Multivariate analyses**				
Stage	1.803 (1.306-2.490)	0.000**	1.686 (1.223-2.324)	0.001**
Lymph node	1.449 (1.060-1.981)	0.020*	1.396 (1.022-1.906)	0.036*
NQO1	1.387 (1.017-1.890)	0.039*	1.154 (0.845-1.576)	0.368

*P < 0.05, **P < 0.01; 95% CI: 95% confidence interval.

## Discussion

Professor Ernster first discovered NQO1 in 1958 [17]. This ubiquitous flavoprotein has been found to be expressed in body tissues [18], and has been localized primarily in the cytoplasm with lower levels being detected in the nucleus [19]. Several functions of NQO1 have been proposed including xenobiotic detoxification, superoxide scavenging, maintenance of endogenous antioxidants, modulation of p53 and proteasomal degradation [20]. It is conceivable that NQO1 is primarily involved in protecting normal cells from oxidant stress and electrophilic attack. These functions have also led to the suggestion that NQO1 plays an important role in cancer chemoprevention. Some studies have shown that the polymorphism in the NQO1 gene affects the translation of the NQO1 protein. The NQO1 C609T polymorphism has been reported to be associated with an increased risk of various cancers such as renal [21], lung [22], esophageal [23], gastric [24] and head and neck [25]. Hu’ results indicated that functional polymorphisms in NQO1 SNP609 associate with the risk of cervical cancer especially in women infected with type 16- and/or type 18-related HPVs [26].

It was also reported that the NQO1 protein and mRNA were abnormally elevated within many solid tumors. Awadallah *et al.*[10] and Lyn-Cook *et al.*[8] found that NQO1 was not only upregulated in pancreatic ductal adenocarcinoma (PDAC), but could also minimize the risk of false positive diagnosis by combining NQO1 expression with cellular morphology assessment. Mikami *et al.*[27] reported a close correlation between NQO1 enzyme activity and protein expression in both colon cancer cell lines and colorectal tumor samples. They also determined that tumors with nodal metastases showed significantly higher NQO1 protein levels than did tumors without metastasis, which suggested that NQO1 expression might be related to tumorigenesis and malignant progression of colorectal tumors.

Our previous study [24] also showed that NQO1 was a significant prognostic or predictive marker in gastric cancer. In this study, we performed IHC staining and analysis of 177 cervical SCC samples, 94 precancerous disease samples and 25 normal epithelium tissues of the uterine cervix, and found that the strongly positive rate of NQO1 protein expression in both SCCs and CINs was significantly higher than in the normal cervix. Interestingly, the strongly positive rate of NQO1 protein was slightly higher in well-differentiated SCC (43.75%) than in CIN3 (40.74%) (P > 0.05), indicating that abnormal NQO1 expression might be an early event in the progression of cervical cancer. In addition, the qRT-PCR result also confirmed an increased level of NQO1 mRNA expression in SCCs compared with normal fresh cervical epithelium tissues.

To further illustrate that NQO1 was a potential effective predictor of poor prognosis, we analyzed the correlation between NQO1 expression and clinicopathological features of cervical SCCs, and found that high-level expression of the NQO1 protein was significantly correlated with poor differentiation, late clinical stage, and the presence of lymph node metastasis (*P* < 0.05). These results indicated that NQO1 played a potentially predictive role in tumor progression of cervical SCCs.

The presence of high-risk HPV infection has been found to be the main cause of cervical cancer [28]. So far, more than 200 HPV types have been reported and many HPVs have been identified in healthy individuals who have no clinical symptoms, while the path from initial infection to severe epithelial lesion still remains unknown [29]. Recent studies suggested that microRNAs have important effects in the manifestation of HPV infections in target epithelial cells [30]. Geiger *et al.*[31] found that during the very early stages of transformation in HPV16-transformed keratinocytes, many epithelial features were gradually eliminated and some mesenchymal traits emerged. In the present study, the positive rate of high-risk HPV infection was 65.52% in CIN-1, 76.32% in CIN-2, 74.07% in CIN-3 and 86.44% in SCC of the cervix, according to ther HPV-DNA chip results. The strongly positive rate of NQO1 protein expression was significantly higher in HPV-positive cervical SCCs (58.17%) than in HPV-negative cases (33.33%). Therefore, it is possible that abnormal expression of NQO1 may make the cervix more prone to HPV infection, and subsequently, HPV infection could accelerate NQO1 overexpression and lead to invasion and metastasis of cervical SCCs [26].

Regarding survival, we found that cervical SCC patients with high-level NQO1 expression had lower DFS (P < 0.01) and 5-year OS rates (P < 0.01) than patients with low-level NQO1 expression. In early-stage cervical SCC, patients with high-level NQO1 expression had lower DFS and 5-year OS rates compared with those with low-level NQO1 expression (P < 0.05, respectively). Moreover, along with clinical stage and lymph node metastasis, multivariate survival analysis demonstrated that NQO1 expression emerged as a significant independent hazard factor for DFS but not for 5-year OS in patients with cervical SCC. These results indicated that NQO1 was a potential predictor of poor prognosis, especially in patients with early-stage cervical SCC.

Overall, our present work implies that NQO1 might be a new biomarker for early diagnosis and prognostic evaluation as well as a potential molecular target in patients with cervical SCC. However, NQO1 was upregulated as a part of the oxidative stress response and inexplicably overexpressed in particular types of tumors, whose function has not yet been elucidated [32]. Marco *et al.*[11] demonstrated that the expression of NQO1 significantly induced cell cycle progression via the upregulation of cyclin A2, B1 and D1 that led to the proliferation of melanoma cells, which may account for the overexpression of NQO1 in primary melanoma. Lau *et al.*[33] postulated that NQO1 overexpression was accompanied by an increase in other antioxidant enzymes, such as HMOX-1 and GST, providing tumors with increased protection against cytotoxic agents allowing for rapid cancer progression.

Recent studies on the regulation of NQO1 gene expression have shown that a complex molecular pathway was involved. In addition, studies of Yao *et al.* showed that induction of the NQO1 gene by hypoxia and mitomycin C in human colon adenocarcinoma HT29 and human hepatoma HepG2 cells was mediated through a mechanism involving the NF-κB signaling pathway [34,35], which has been shown to play an important role in proliferation, resistance to apoptosis, invasion, and metastasis of HeLa cells [36,37]. Even in light of this recent information, the molecular mechanism of NQO1 responsible for cervical tumor progression remains to be elucidated, and additional studies are warranted to further our understanding of the role that NQO1 plays in cervical tumorigenesis.

## Conclusions

NQO1 plays an important role in the tumorigenesis and might be an independent biomarker for prognostic evaluation in cervical SCCs.

## Competing interests

The authors declare that they have no competing interests.

## Authors’ contributions

YM, JK, TJ and DJ participated in the study conception, design, case selection and experiments. GY, XR and YM carried out data collection. TJ, LL and ZL performed the data analysis and wrote the manuscript. All authors read and approved the final manuscript.

## Pre-publication history

The pre-publication history for this paper can be accessed here:

http://www.biomedcentral.com/1471-2407/14/414/prepub

## Supplementary Material

Additional file 1: Table S1 NQO1 protein expression in cervical SCC. **Table S2.** Correlation between HPV infection and NQO1 expression in cervical lesions. Click here for file
