# Supplementary material for: NQO1 overexpression is associated with poor prognosis in squamous cell carcinoma of the uterine cervix
Source: BMC Cancer. 2014 Jun 9;14:414. doi: 10.1186/1471-2407-14-414 (PMC4058702; doi:10.1186/1471-2407-14-414)
Supplement: Additional file 1: Table S1 — NQO1 protein expression in cervical SCC. Table S2. Correlation between HPV infection and NQO1 expression in cervical lesions. [file 1471-2407-14-414-S1.doc]

**Table S1:** NQO1 protein expression in cervical SCC.

| **Diagnosis** | **No. of cases** | **NQO1 protein expression** | **Positive rate** | **Strongly positive rate** |
| --- | --- | --- | --- | --- |
|  |  | **- + ++ +++** |  |  |
| Normal cervix | 25 | 22 2 1 0 | 12.00% | 4.00% |
| CIN-1 | 29 | 17 4 6 2 | 41.38%* | 27.59%* |
| CIN-2 | 38 | 18 7 8 5 | 52.63%** | 34.21%** |
| CIN-3 | 27 | 12 4 5 6 | 55.56%** | 40.74%** |
| SCC | 177 | 35 45 56 41 | 80.23%** | 54.80%** |

**P*<0.05 and ***P*<0.01: compared with normal cervical epithelial tissues.

CIN: Cervical intraepithelial neoplasia; SCC: Squamous cell carcinoma

**Table S2:** Correlation between HPV infection and NQO1 expression in cervical lesions.

| **Diagnosis** | **Total *n*** | **Case *n* (%) of HPV status** | **Strongly NQO1 positive rate (%)** |
| --- | --- | --- | --- |
|
| **Normal cervix**  HPV +  HPV - | 25 | 0 (0.00%)  25 (100%) | 0 (0.00%)  1 (4.00%) |
| **CIN-1**  HPV +  HPV - | 29 | 19 (65.52%)  10(34.48%) | 6 (31.58%)  2 (20.00%) |
| **CIN-2**  HPV +  HPV - | 38 | 29 (76.32%)  9 (23.68%) | 10 (34.48%)  3 (33.33%) |
| **CIN-3**  HPV +  HPV - | 27 | 20 (74.07%)  7 (25.93%) | 9 (45.00%)  2 (28.57%) |
| **SCC**  HPV +  HPV - | 177 | 153 (86.44%)  24 (13.56%) | 89 (58.17%)*  8 (33.33%) |

**P*=0.023. CIN: Cervical intraepithelial neoplasia; SCC: Squamous cell carcinoma
